# Adaptive Machining Method for Helical Milling of Carbon Fiber-Reinforced Plastic/Titanium Alloy Stacks Based on Interface Identification

**DOI:** 10.3390/ma17020297

**Published:** 2024-01-07

**Authors:** Chao Yan, Renke Kang, Fantong Meng, Zhigang Dong, Yan Bao, Guolin Yang

**Affiliations:** State Key Laboratory of High-Performance Precision Manufacturing, Dalian University of Technology, Dalian 116024, China

**Keywords:** CFRP/Ti stacks, helical milling, interface identification, adaptive method

## Abstract

CFRP/Ti stacks composed of carbon fiber-reinforced plastic composites (CFRP) and titanium alloys (Ti) are widely used in aerospace fields. However, in the integrated hole-making process of CFRP/Ti stacks, the machining characteristics of various materials are significantly different, and constant machining parameters cannot simultaneously meet the high-quality machining requirements of two materials. In addition, errors exist between the actual thickness of each material layer and the theoretical value, which causes an impediment to the monitoring of the machining interface and the corresponding adjustment of parameters. An adaptive machining method for the helical milling of CFRP/Ti stacks based on interface identification is proposed in this paper. The machining characteristics of the pneumatic spindle and the interface state in the helical milling of CFRP/Ti stacks are analyzed using self-developed portable helical milling equipment, and a new algorithm for the real-time monitoring of the machining interface position and adaptive adjustment of the machining parameters according to the interface identification result is proposed. Helical milling experiments were carried out, the results show that the proposed method can effectively identify the position of the machining interface with good identification accuracy. Moreover, the proposed parameter-adaptive optimized machining method for CFRP/Ti stacks can significantly improve hole diameter accuracy and machining quality.

## 1. Introduction

Assembly is a very important part of the aerospace manufacturing field, and the main combination form of various structural components in the assembly process is the bolted and riveted connection. Millions of bolt and rivet holes may need to be manufactured at the assembly site to fasten different materials and their components together in order to build an airplane, which makes the machining of connecting holes an indispensable process in aerospace assembly. The fatigue strength of assembled structural components is closely related to the quality of the machined holes [1,2,3,4], which shows that the precision and quality of the hole-making process will directly affect the assembly effect and subsequent flight safety [5,6]. Carbon fiber-reinforced plastic composites (CFRP) are very commonly, heavily, and universally used advanced composites in aerospace assembly, processing, and manufacturing, which have a strong representation. Titanium alloys (Ti) also have excellent material properties, and CFRP/Ti stacks composed of the two materials together exhibit excellent mechanical properties and flexible designability, which have been widely used in the manufacturing of components at critical positions of aircraft, providing an important guarantee for the safety, durability, and environmental protection of aircraft [7,8,9,10]. Stacked components need to be assembled by riveting or bolting after the hole-making process. The tolerance requirements of the hole are more stringent based on the consideration of safety factors [11,12], which has made the problem of ensuring a high-precision and high-quality hole-making process for CFRP/Ti stacks become the focus of attention in much of the research.

The conventional drilling method is commonly used in the hole-making process of CFRP/Ti stacks in the aerospace field, which accounts for 40–60% of the total material removal process, with high axial force, difficult chip removal, and poor heat dissipation. In addition, the hole-making process may include multiple procedures such as drilling, expanding, and reaming depending on the machining requirements, which further increases the cost of cutting tools [13,14,15,16]. Helical milling is an emerging hole-making method in the aerospace field, in which a specialized cutting tool rotates around its own axis while feeding along a helical trajectory. Compared with conventional drilling, the helical milling method has significant advantages in terms of hole-making accuracy and quality, cutting tool service life, and applicable machining objects, and has been used in the hole-making process of difficult-to-machine materials in aerospace [17,18,19]. The physical and mechanical properties of the two materials in CFRP/Ti stacks are different, which makes it difficult to ensure hole-making quality and efficiency [20,21], so the helical milling method for CFRP/Ti stacks has been widely studied. Several studies analyzed the kinematics of helical milling, concentrating on cutting forces and cutting heat during the machining process [22,23,24]. In addition, the influence of parameters on the machining quality during helical milling was also thoroughly investigated [25,26]. Some researchers carried out a comparative analysis of drilling and helical milling in composites and metal stacks, and the results showed that the helical milling method has lower cutting forces and machining temperatures, higher machining accuracy, and significantly lower surface roughness and machining burrs [27,28]. Zhou et al. [29] analyzed the effects of spindle speed and the hole-tool diameter ratio on hole diameter error and roundness error during the helical milling of CFRP/Ti stacks. He et al. [30] carried out an experimental study on the helical milling of CFRP/Ti-6Al-4V stacks using constant parameter and variable parameter machining strategies, respectively. The results showed that longer tool life, lower cutting forces, and better machining quality were achieved with a variable parameter strategy. Wang et al. [31] carried out experimental research on helical milling using two machining strategies for the processing sequence of CFRP/Ti stacks and analyzed the effects of different machining strategies and the order of the stacked materials on the cutting forces, cutting temperature, and machining quality of the holes.

The material properties and machining characteristics of the different materials in the CFRP/Ti stack are significantly different from each other, so each layer has its own suitable machining parameters. Furthermore, the thickness of each material layer at different hole-making positions may not be uniform due to the requirements of the shape of the component, and there is a certain error between the actual thickness and the theoretical value, which makes it difficult to accurately control the machining position and adjust the machining parameters promptly. Pan et al. [32] proposed an online monitoring method for the position of the helical milling of CFRP/Ti stacks based on a robotic hole-making system. Neugebauer et al. [33] utilized acoustic emission signals to monitor the real-time position of the cutting tool during manufacturing. The above research methods either require extra sensors or rely on large hole-making systems for support, and none of the studies have investigated the optimization of the processing parameters after monitoring the machining position.

In this paper, a new adaptive machining method for the helical milling of CFRP/Ti stacks based on interface identification is proposed to solve the above problems. The machining principle of the helical milling process is analyzed. The portable helical milling equipment is developed independently, which is not only suitable for conventional machining sites but also effectively solves the problem of poor environmental openness of certain machining locations in practical industry applications. Using the independently developed portable helical milling equipment, the machining characteristics of pneumatic spindle speed varying with torque and the machining interface state of CFRP/Ti stacks in the helical milling process are investigated, and the corresponding relationship between spindle speed and different machining stages is established. A new algorithm for the real-time monitoring of the position of the machining interface and adaptive adjustment of the machining parameters based on the interface identification results is proposed, which enables high-quality optimization of the CFRP/Ti stack hole-making process. Helical milling experiments are carried out, and the results show that the proposed interface identification method is not sensitive to the thickness uniformity and manufacturing errors at each place of the stacks, and it can effectively determine the positions of the machining interfaces with good identification accuracy. In addition, the parameter adaptive machining method for the helical milling of CFRP/Ti stacked layers based on interface identification can significantly improve the hole diameter accuracy, interlayer hole diameter consistency, and machining quality, which verifies the advancement and superiority of the proposed method. Moreover, the study content of this paper is tested in some domestic aerospace assembly and manufacturing enterprises, which is of great practical application value and significance for the optimization of the hole-making process of CFRP/Ti stacks in actual product manufacturing.

## 2. Materials and Methods

### 2.1. Machining Principle of the Helical Milling Method

**Figure 1:**
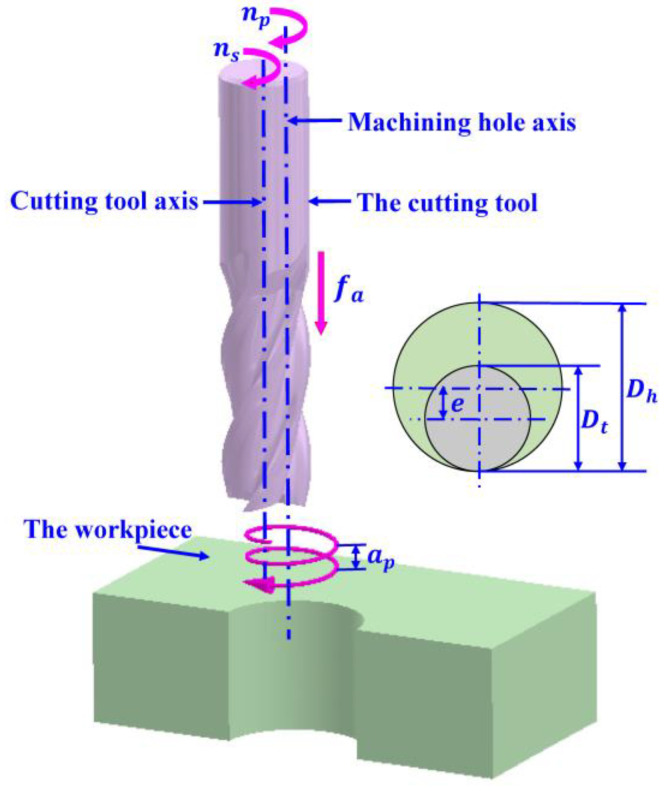
Machining principle of helical milling.

During the helical milling process, the bottom and side edges of a special end mill are involved in the cutting process at the same time. The main cutting motion is realized by the cutting tool rotating along its own axis with the speed *n_s_* (r/min), which is called the rotation motion. At the same time, the whole cutting tool rotates along the axis of the hole to be machined with the speed *n_p_* (r/min), which is called the revolution motion. In addition to the above two motions, the cutting tool is also fed along its own axis with the speed *f_a_* (mm/min), which is called the axial feed motion. The above three motions are carried out simultaneously to form a complete motion trajectory for helical hole milling, as shown in Figure 1.

The helical milling method is able to machine a hole with a diameter larger than the diameter of the cutting tool. The diameter of the cutting tool is defined as *D_t_*, the diameter of the hole is defined as *D_h_*, and the distance between the axis of the cutting tool and the axis of the machined hole is defined as the eccentricity distance *e*. Then, there is a relationship as follows:

(1) $$D_{h}=D_{t}+2e$$

The revolution motion is combined with the axial feed motion to form the helical trajectory motion of the cutting tool, for which the helical pitch *a_p_* can be expressed as follows:

(2) $$a_{p}=\frac{f_{a}}{n_{p}}$$

The helical feed velocity of the cutting tool along the hole axis is called *f* (mm/min), which can be obtained by combining the tangential feed velocity *f_t_* and the axial feed velocity *f_a_*:

(3) $$f_{t}=\pi n_{p}\left(D_{h}-D_{t}\right)$$

(4) $$f=\sqrt{{\left(f_{t}\right)}^{2}+{\left(f_{a}\right)}^{2}}$$

The helical rise angle *α* of the helical trajectory of the tool is expressed as follows:

(5) $$\alpha =\arctan\frac{f_{a}}{f_{t}}$$

The tangential feed per tooth *f_zt_* (mm/tooth) and axial feed per tooth *f_za_* (mm/tooth) of the cutting tool are expressed as follows:

(6) $$f_{zt}=\frac{f_{t}}{N\cdot n_{s}}$$

(7) $$f_{za}=\frac{f_{a}}{N\cdot n_{s}}$$

where *N* denotes the number of teeth on the cutting tool.

### 2.2. Adaptive Method Based on Interface Identification

#### 2.2.1. Portable Helical Milling Equipment

**Figure 2:**
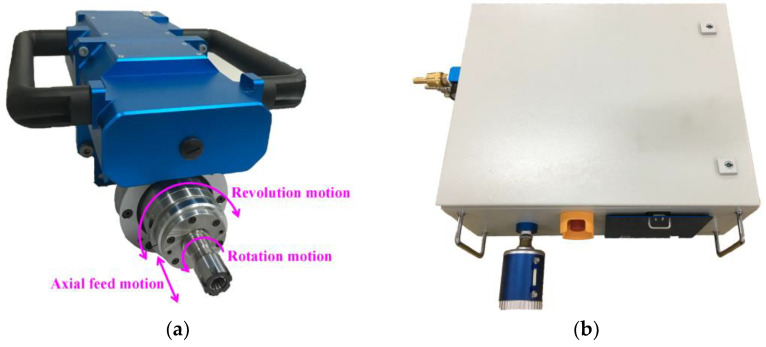
The portable helical milling equipment. (**a**) Portable helical milling implementation unit. (**b**) Control box of the equipment system.

Targeting at the demands for high-quality hole-making of stacks made of difficult-to-machine materials in the aircraft and aerospace field, as well as the problems of poor environmental openness of the machining location, the portable helical milling equipment (Dalian University of Technology, Dalian, China) in this study was developed independently. As shown in Figure 2, the equipment has the technical advantages of miniaturization and lightweighting, is able to provide the motion required for helical milling, and all the motion accuracy indexes meet the requirements of aerospace component assembly and hole-making.

**Figure 3:**
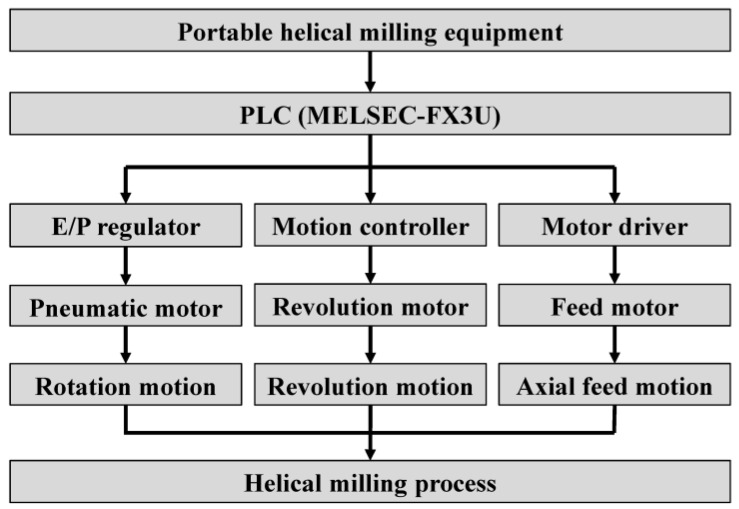
The principle of the motion control system.

The center of the self-designed and built control system for the portable helical milling equipment is the MELSEC-FX3U series PLC (MITSUBISHI ELECTRIC, Tokyo, Japan), which controls the pneumatic motor (Ober, Italy), revolution DC motor (FAULHABER, Schönaich, Germany), and feed stepping motor (Oriental motor, Tokyo, Japan) by sending commands to the E/P regulator (SMC, Tokyo, Japan), motion controller, and motor driver, thus achieving the rotation motion, revolution motion and axial feed motion in the helical milling process. The equipment performs the functions of center cooling, automatic tool setting, multi-functional and multi-step automated hole-making, and so on. The hole-making accuracy, quality, and machining efficiency can satisfy all requirements, and the machining adaptability is excellent. The principle of the motion control system is shown in Figure 3.

**Figure 4:**
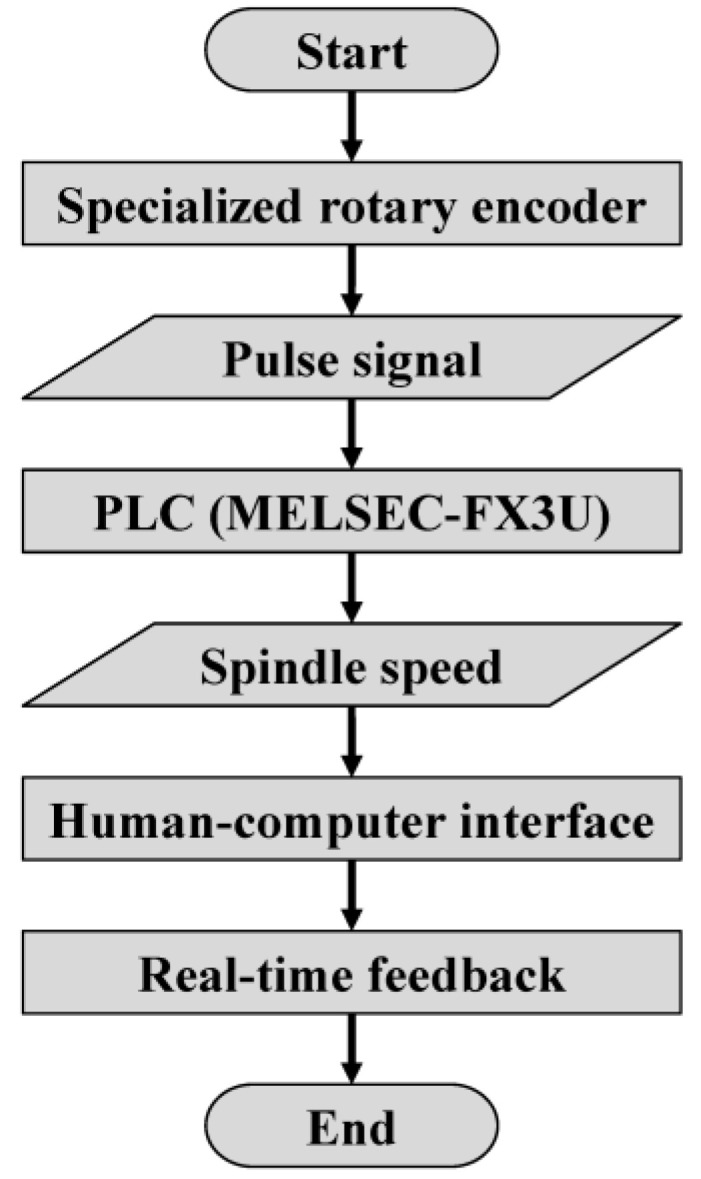
Spindle speed real-time feedback monitoring process.

The spindle speed real-time feedback monitoring module of the equipment was designed and developed, and the operation process is shown in Figure 4. The pulse signal is sent from the rotary encoder (OMRON, Kyoto, Japan) to the PLC. Then, the current spindle speed value is obtained with arithmetic processing, transmitted to the output module in real time, and displayed on the developed human–computer interface. This module can realize the real-time reading of the spindle speed and the monitoring of the machining status of the equipment, providing an important basis for the identification and judgment of the machining interface.

#### 2.2.2. Machining Characteristics of the Pneumatic Spindle

The pneumatic motor has the advantages of being lightweight, having a low working temperature, and easy maintenance, which can meet the design requirements of the equipment. The output shaft of the pneumatic motor is connected to the input shaft of the rear end of the mechanical spindle through a coupling to compose a pneumatic spindle, thus providing the rotation motion of the spindle. The adjustment of the spindle speed is realized by controlling the E/P regulator.

The mathematical relationship between power (*P*), torque (*T*), and speed (*n_s_*) of the pneumatic spindle can be expressed as follows:

(8) P=FV

(9) F=T/R

(10) $$V=\frac{2\pi Rn_{s}}{60}$$

In the above equation, *F* is the torsion force, *V* is the linear velocity, and *R* is the torsion radius. Bringing Equations (9) and (10) into Equation (8) results in the following equation:

(11) $$P=\frac{\pi Tn_{s}}{30}$$

**Figure 5:**
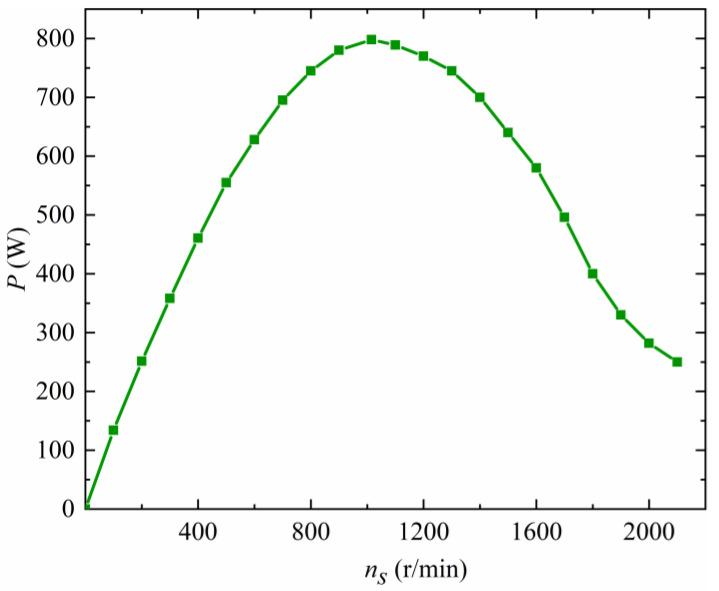
Power–speed relationship of the pneumatic spindle.

The power–speed relationship curve of the pneumatic spindle of the equipment at its rated input air pressure is shown in Figure 5. By bringing the corresponding power and spindle speed values into Equation (11) for calculation and analysis, it is found that the spindle speed of the pneumatic spindle decreases with an increase in the torque applied to the spindle for a constant input air pressure.

#### 2.2.3. Interface Identification and Adaptive Machining Principles

Due to the significant difference in the material characteristics and machinability of CFRP and Ti, the cutting forces and torques applied to the cutting tool in the process of the helical milling of CFRP/Ti stacks are different when the cutting tool is machining at the CFRP entry interface, the CFRP/Ti transition interface, and the Ti exit interface. The pneumatic spindle of the self-developed portable helical milling equipment has the characteristic that the spindle speed decreases with an increase in torque when the input air pressure is certain, so the spindle speed of the equipment will change in different magnitudes when machining at the different material interfaces. Based on the above analysis, this paper proposes an adaptive machining method for the helical milling of CFRP/Ti stacks based on interface identification.

**Figure 6:**
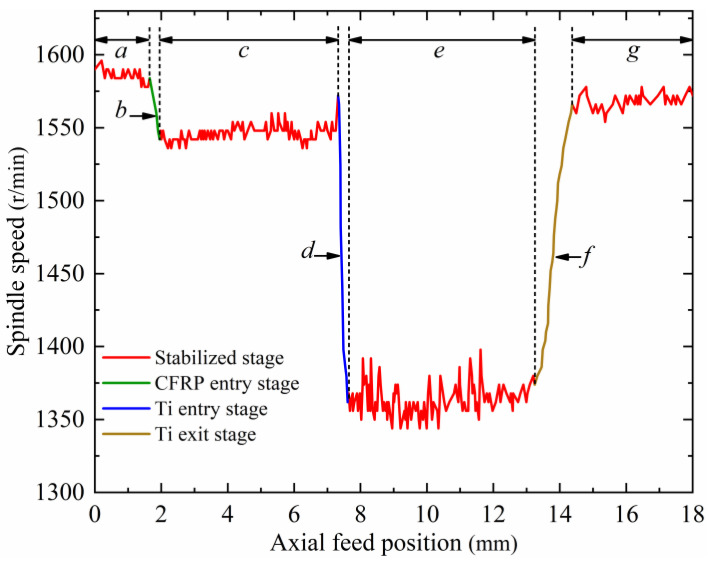
Spindle speed–axial feed position curve. (The explanations for the letter a-g are as follows).

Firstly, a single-parameter helical milling experiment for CFRP/Ti stacks was carried out on the self-developed portable helical milling equipment. The experimental material is a CFRP/Ti-laminated flat plate, in which the fiber filament grade chosen for the CFRP is T800, and the material thickness is 5.5 mm; the titanium alloy is chosen as Ti-6Al-4V (TC4), and the material thickness is 6.5 mm. The cutting tool used for the experiment is the 4-edge end mill, the tool material is cemented carbide, and the target hole diameter is 12 mm. The initial machining parameters are: *n_s_* = 1600 r/min, *n_p_* = 22 r/min, and *f_a_* = 10 mm/min. The experiment was repeated four times, and the average value was taken as the spindle speed reference value. Next, the designed and developed spindle speed real-time feedback module was utilized to extract the relationship curve between the spindle speed and the change in the axial feed position during the machining process using data operations and processing, as shown in Figure 6.

**Figure 7:**
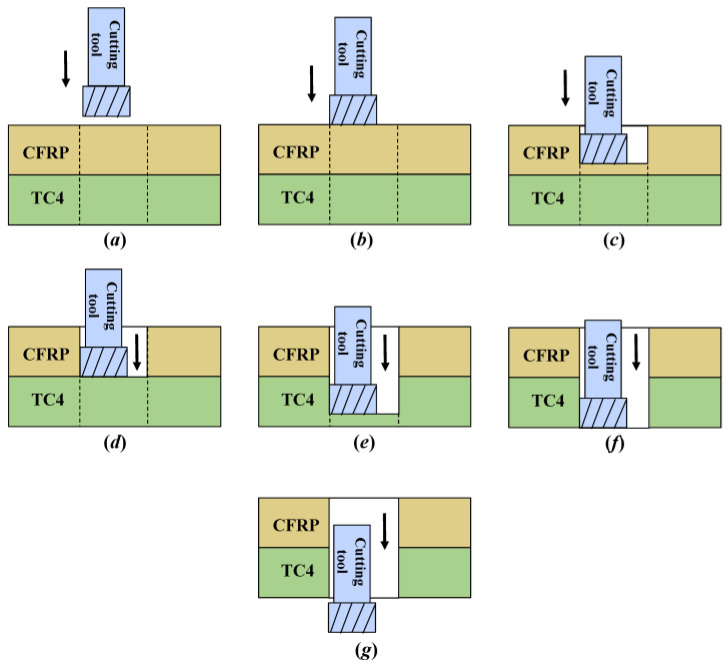
Helical milling stage of CFRP/Ti stacks.

The helical milling state corresponding to each machining stage in Figure 6 is shown in Figure 7. Using analysis, it is found that the whole helical milling process can be divided into seven stages. In stage *a*, the equipment is started and fed forward according to the set parameters; at this time, the cutting tool has not yet contacted the workpiece. In stage *b*, the cutting tool processes to the CFRP entry interface, the torque applied increases, and the spindle speed consequently generates a significant decrease. In stage *c*, the cutting tool machines the CFRP, and the torque applied is stable; therefore, the spindle speed is relatively steady. In stage *d*, the cutting tool processes to the CFRP/Ti transition interface, the torque applied increases further, and the spindle speed decreases considerably. In stage *e*, the cutting tool machines Ti, and due to the difficult-to-machine property of the material, the spindle speed has some fluctuation but is overall smooth. In stage *f*, the cutting tool processes to the Ti exit interface and the torque applied decreases; therefore, the spindle speed increases. In stage *g*, the cutting tool completely cuts out of the Ti, and the hole-making process ends.

**Figure 8:**
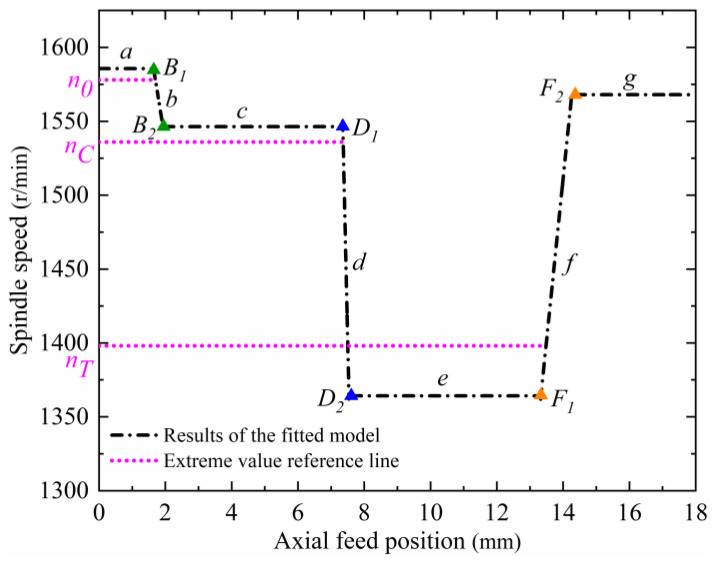
Spindle speed–axial feed position linear fitting result.

Within stages *b*, *d*, and *f*, the threshold values of spindle speed variation, *n_Ce_*, *n_Te_*, and *n_To_*, are extracted using computational analysis and are used as the basis for the identification and judgment of the CFRP entry interface, CFRP/Ti transition interface, and Ti exit interface. Due to the fluctuation in the cutting forces and torque applied to the cutting tool during the machining process, the real-time spindle speed also fluctuates. In order to eliminate the influence of spindle speed fluctuation and improve the accuracy of the selected threshold values, a fitting model for the relationship between spindle speed and axial feed position is established. Firstly, the spindle speed–axial feed position curve is linearly fitted in stages using the least squares method, as shown in Figure 8, in which *B*_1_, *B*_2_, *D*_1_, *D*_2_, *F*_1_, and *F*_2_ are the starting and ending points of the spindle speed change when the cutting tool processes to the CFRP entry interface (stage *b*), the CFRP/Ti transition interface (stage *d*), and the Ti exit interface (stage *f*), respectively. *n*_0_ is the reference minimal value of the spindle speed in stage *a*, *n_C_* is the reference minimal value of the spindle speed in stage *c*, and *n_T_* is the reference maximal value of the spindle speed in stage *e*.

The expression of the linear fitting model for machining stages *a~g* is given below:

(12) $$y_{a\sim g}=K_{a\sim g}\cdot x+N_{a\sim g}$$

The parameters of the model for each machining stage are shown in Table 1.

Figure 8 shows that if the threshold value is selected too close to the starting point of the spindle speed change at the interface (*B*_1_, *D*_1_, *F*_1_), the spindle speed change is not obvious at that time, and it may not be able to accurately identify the interface position. If the threshold value is selected too close to the ending point of the spindle speed change at the interface (*B*_2_, *D*_2_, *F*_2_), the identified interface position will have a large hysteresis compared with the actual position. In addition, the determination of the identification threshold value for each interface should also satisfy the following mathematical relationship with the spindle speed extreme value of its previous stage: *n_Ce_* < *n*_0_, *n_Te_* < *n_C_*, *n_To_* > *n_T_*. Based on the above constraint conditions, and considering the influence of interface identification accuracy and hysteresis, the spindle speed value at 0.02 mm backward from the reference extreme value position of each machining stage is finally selected as the threshold value for the identification of each interface. The set reference threshold values are obtained by substituting them into the linear fitting model, as shown in Table 2.

**Figure 9:**
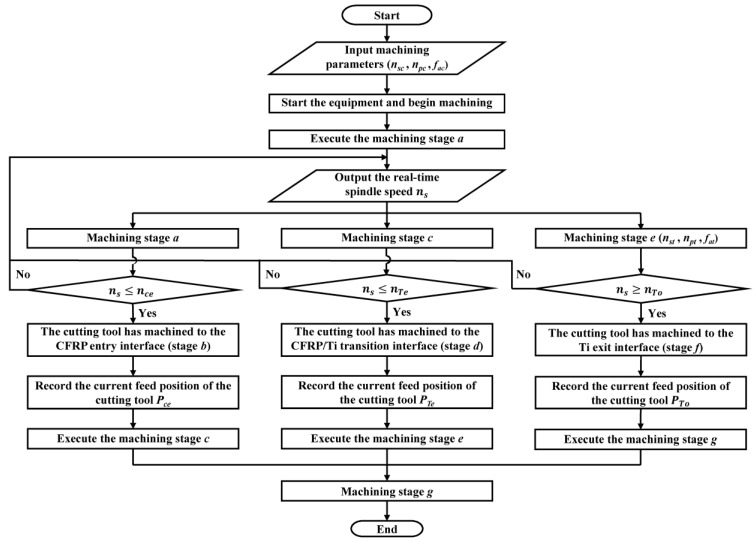
The adaptive machining algorithm process based on interface identification.

The algorithm process of the adaptive machining method for helical milling of CFRP/Ti stacks based on interface identification is shown in Figure 9. First, the initial machining parameters (*n_sc_*, *n_pc_*, *f_ac_*) are input. At this time, the machining parameters used are suitable for the stable machining of CFRP, and the equipment starts to work. Next, the cutting tool machining is executed in stage *a*. At this time, the cutting tool has not yet touched the CFRP, and the spindle speed is fed back and output to the PLC in real time. When the spindle speed *n_s_* ≤ *n_Ce_*, the cutting tool is considered to have processed to the CFRP entry interface (stage *b*), at which time the current feed position *P_Ce_* of the cutting tool is recorded. Then, the cutting tool machining stage *c* is executed, with the machining parameters remaining unchanged. When the spindle speed *n_s_* ≤ *n_Te_*, the cutting tool is considered to have processed to the CFRP/Ti transition interface (stage *d*), at which time the current feed position *P_Te_* of the cutting tool is recorded. Then, cutting tool machining stage *e* is executed, and at this time, the machining parameters are adaptively adjusted to the parameter combinations that are suitable for the stable machining of Ti (*n_st_*, *n_pt_*, *f_at_*). When the spindle speed *n_s_* ≥ *n_To_*, the cutting tool is considered to have processed to the Ti exit interface (stage *f*), and at this time, the current feed position *P_To_* of the cutting tool is recorded. Finally, the cutting tool machining stage *g* is executed until the tool completely cuts out of Ti. At this time, the adaptive machining algorithm process based on interface identification is finished.

## 3. CFRP/Ti Stack Helical Milling Experiment

### 3.1. Construction of the Experimental Platform

**Figure 10:**
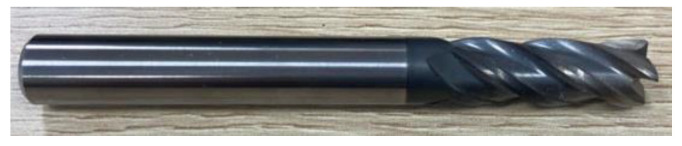
Four-edge end mill used for the experiment.

In order to verify the interface identification accuracy as well as the machining quality and accuracy of the proposed method in this paper, the developed portable helical milling equipment is used to build an experimental platform to carry out a CFRP/Ti stack helical milling validation experiment. The experimental material is a CFRP/Ti-laminated flat plate, in which the fiber filament grade chosen for CFRP is T800, and the material thickness is 5.5 mm; titanium alloy is chosen as Ti-6Al-4V (TC4), and the material thickness is 6.5 mm. The cutting tool used for the experiment is the four-edge end mill (MISUMI, Tokyo, Japan), and the tool material is cemented carbide, as shown in Figure 10.

**Figure 11:**
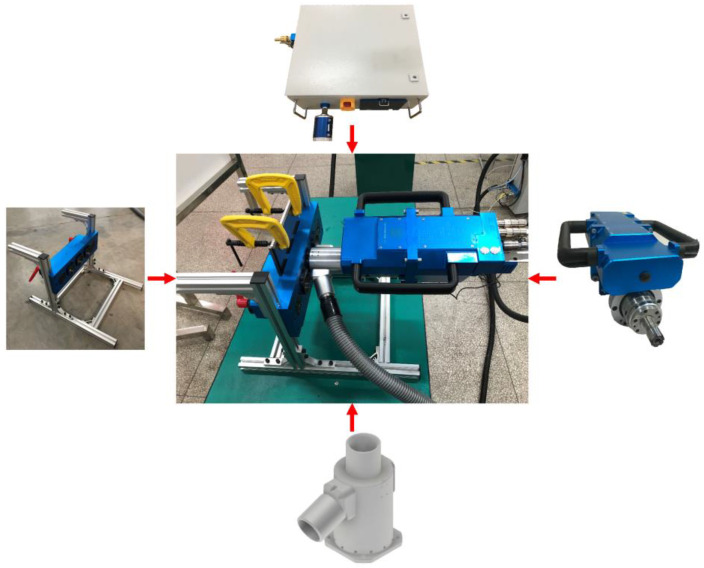
The helical milling experimental platform.

The helical milling experimental platform is shown in Figure 11. The material to be machined is clamped on a special drilling template, and the portable helical milling equipment is assembled and fixed to the drilling template using a docking sleeve. Under dry-cutting conditions, an external dust suction tube is used to help discharge the chips. The diameters of the machined holes in the experiment were measured using a Mitutoyo HTD-20RST (Mitutoyo, Kanagawa, Japan) three-jawed inside micrometer, the surface roughness of the hole wall was measured using a Mitutoyo SJ-210 portable roughness gauge, (Mitutoyo, Kanagawa, Japan) and the entrance/exit quality of the machined holes were observed using an INSIZE ISM-DL301-Y microscope (INSIZE, Suzhou, China).

### 3.2. Design of the Experiment

The CFRP was placed in the upper position of the stacks, which first contacted the cutting tool, and the TC4 was put in the lower position of the stack. The target diameter of the machined hole for the helical milling experiment is 12 mm. The machining parameter combination suitable for the CFRP (*n_sc_*, *n_pc_*, *f_ac_*) and the machining parameters combination suitable for the TC4 (*n_st_*, *n_pt_*, *f_at_*) were selected. Two machining experimental methods were designed: the first one uses the machining parameters suitable for CFRP to perform constant parameter helical milling of the entire CFRP/Ti stack, and the second one utilizes the parameters combination suitable for the CFRP and TC4 layers, respectively, for the validation of the adaptive machining method for the helical milling of CFRP/Ti stacks based on interface identification. The experimental machining parameters are shown in Table 3. The number of holes made for each experimental method was six, the experiment was repeated three times, and the average value was taken as the experimental result.

## 4. Results and Discussion

### 4.1. Accuracy of Interface Identification

**Figure 12:**
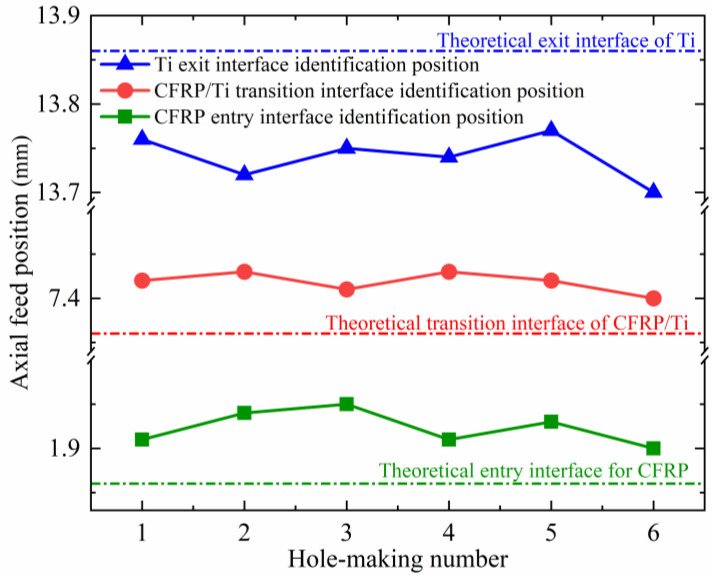
Relative relationship between the interface identification position and the theoretical interface.

Using the proposed interface identification method, the real-time feed positions *P_ce_*, *P_Te_*, and *P_To_* fed back by the equipment system when the cutting tool is machining into the CFRP entry interface, the CFRP/Ti transition interface, and the Ti exit interface were recorded in the experiment. The error between the interface position monitored with the interface identification method and the theoretical machining interface position of the workpiece obtained with the calculation is shown in Table 4, where a positive number indicates that the position monitored with the interface identification method lags behind the theoretical position, and a negative number indicates that the monitored position is in advance of the theoretical position. The results show that at the entry interface of the CFRP, the identification errors do not exceed 0.09 mm. At the transition interface of the CFRP and TC4, the identification errors do not exceed 0.07 mm. For the exit interface of the TC4, the identification errors are 0.09~0.16 mm, and the interface monitoring position is ahead of the theoretical position. This is because during the hole-making process of the TC4 layer, as the thickness of the unprocessed material becomes smaller and smaller, the cutting forces and torque applied to the cutting tool are consequently reduced, causing the spindle speed of the pneumatic spindle to gradually increase, resulting in the spindle speed exceeding the set threshold value before the time the TC4 is finished machining, and thus, the identified position of the interface will shift forward with respect to the theoretical position. The relative relationship between the monitoring interface position derived from the interface identification method and the theoretical machining interface position is shown in Figure 12.

In other studies, Pan et al. [32] proposed an online monitoring method for the machining position of the helical milling of CFRP/Ti stacks based on a robotic hole-making system. Comparing the interface identification results obtained in this study with the previous relevant study by Pan et al. [32], it is found that the accuracy of interface identification for the CFRP entry interface and transition interface obtained in this study is slightly higher. For the TC4 exit interface, the interface identification accuracy obtained in this study is significantly higher, and the interface identification positions obtained are ahead of the theoretical positions at the TC4 exit interface in both this study and the relevant study by Pan et al. [32]. Neugebauer et al. [33] utilized acoustic emission signals to monitor the real-time position of the cutting tool during the drilling of CFRP/Al stacks. Compared with that study, the CFRP/Ti stacks studied in this paper have a higher degree of difficult machinability and are now being used increasingly widely. Moreover, the helical milling method used in this paper is an emerging machining technology, which also has its own advantages. The above studies either need to use additional sensors for observation or rely on the support of large-scale hole-making systems. In addition, none of the studies investigated the automatic optimization of the processing parameters after identifying the machining position, which further demonstrates the innovation and advancement of this paper’s research content.

### 4.2. Hole Diameter Accuracy

**Figure 13:**
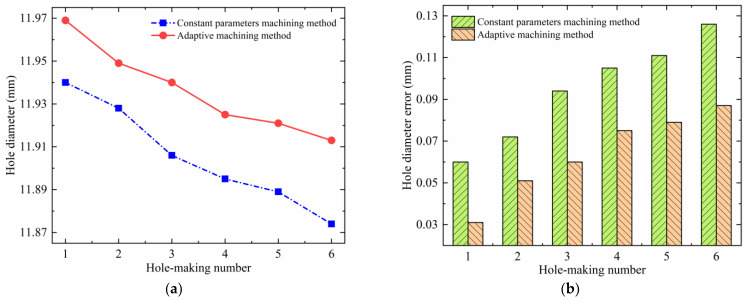
Hole diameter error of the CFRP under two experimental methods: (**a**) hole diameter; and (**b**) hole diameter error.

**Figure 14:**
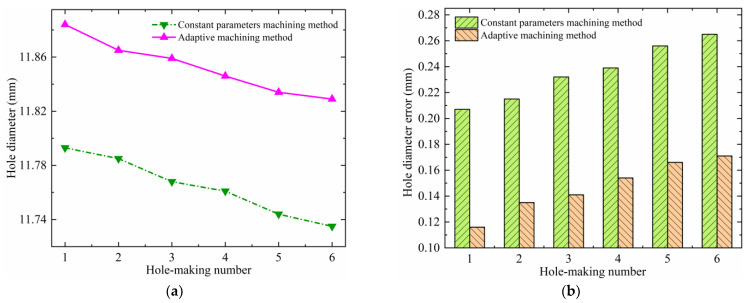
Hole diameter error of the TC4 under two experimental methods: (**a**) hole diameter and (**b**) hole diameter error.

The hole diameters and their errors for the helical milling of CFRP/Ti stacks under the two experimental methods are shown in Figure 13 and Figure 14. Analyzing Figure 13, it is found that although the same parameters are used for machining the CFRP layer under the two experimental methods, the adaptive machining method based on interface identification (abbreviated as the adaptive machining method in the following figures) results in a smaller hole diameter error and a higher hole-making accuracy. The reason is that the constant parameter machining method uses the same parameter combination (*n_sc_*, *n_pc_*, *f_ac_*) to process whole stacks, and these parameters are not suitable for TC4, which leads to serious heat generation of the TC4 layer in the machining process. When the heat is transferred to the CFRP layer, it will further exacerbate the shrinkage and rebound of processed holes, which will have an adverse effect on the hole-making accuracy. Moreover, in this case, the wear and chip adhesion conditions of the cutting tool during the machining of TC4 are more serious, which therefore also causes an enlargement in the CFRP hole diameter error and a reduction in accuracy during the continuous hole-making process. For TC4, the hole diameter accuracy obtained using the adaptive machining method based on interface identification is also significantly higher, as shown in Figure 14. In this method, the equipment is able to accurately identify the position of the cutting tool contacting the CFRP/Ti transition interface and adaptively adjust to the machining parameters (*n_st_*, *n_pt_*, *f_at_*) suitable for TC4. Therefore, the wear, heat, and vibration of the cutting tool are significantly improved compared with the constant parameter machining method, resulting in a smaller hole diameter error and higher accuracy.

**Figure 15:**
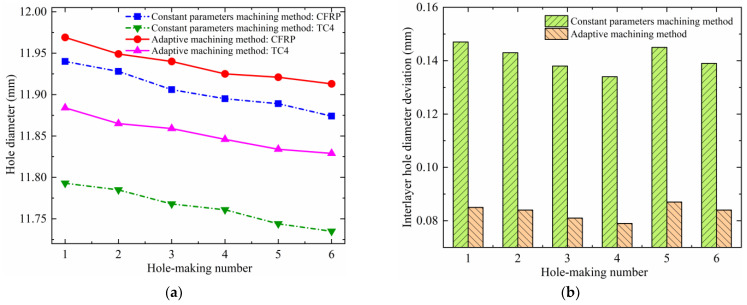
Interlayer hole diameter deviation of the CFRP/Ti stacks using two experimental methods: (**a**) hole diameter and (**b**) interlayer hole diameter deviation.

The actual hole diameter difference between the layers of the CFRP/Ti stacks at the same machined hole location is defined as the interlayer hole diameter deviation. Figure 15 shows the CFRP/Ti stacks’ hole diameters and their interlayer hole diameter deviations under the two machining experimental methods. It was found that the hole diameters of the TC4 layer are smaller than the hole diameters of the CFRP layer in the same group no matter which experimental method is used. Using a comparative analysis, the same phenomenon in the results of this study was also found in the previous related studies by He et al. [30] and Wang et al. [34]. The reason for this is that when processing CFRP/Ti stacks with helical milling, the hole-making sequence of the cutting tool is from CFRP to TC4, and the cutting tool is already subjected to obvious wear when processing to CFRP at first, so the wear of the cutting tool will be more serious when it continues to process to the TC4 layer. In addition, due to the influence of the machinability of different materials, the cutting force applied to the cutting tool is much higher during the machining of TC4, which makes the giveaway phenomenon of the cutting tool more prominent; therefore, the hole diameter of the TC4 layer will be smaller than that of the CFRP layer. The interlayer hole diameter deviation obtained using the adaptive machining method based on interface identification is smaller than that of the constant parameter machining method, suggesting that the former has better interlayer hole diameter consistency. The reason for this is that the machining parameters (*n_st_*, *n_pt_*, *f_at_*) of the lower layer when the adaptive machining method based on interface identification is used are suitable for TC4; therefore, the tool is in a more favorable machining condition and state, which contributes to an improvement in the interlayer hole diameter consistency.

### 4.3. Hole Machining Quality

**Figure 16:**
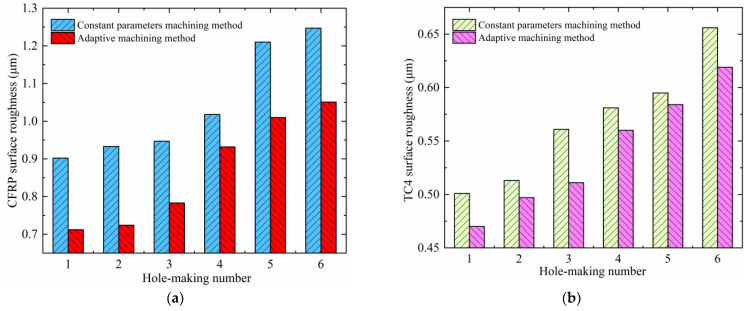
The hole wall surface roughness of the CFRP and TC4 using two experimental methods: (**a**) CFRP and (**b**) TC4.

Using surface roughness as an indicator can be more intuitive, specific, and accurate when assessing the quality of the hole surface, which also makes the comparative analysis much clearer. The surface roughness of the hole wall of the CFRP and TC4 under the two machining methods is shown in Figure 16. A comprehensive analysis shows that using the two test methods, the hole wall surface roughness of the CFRP layer is bigger than that of the TC4 layer. By comparing the results of this study with the previous study by He et al. [30], it is found that the results show similar trends and regularities. The hole wall surface roughness of the CFRP layer is less than Ra1.6, and the hole wall surface roughness of the TC4 layer is less than Ra0.8, which are in line with the standard requirements of machining surface roughness for practical engineering applications. The hole wall surface roughness using the constant parameter machining method is in the range of 0.90~1.25 μm for CFRP and 0.50~0.66 μm for TC4; the hole wall surface roughness using the adaptive machining method based on interface identification is in the range of 0.71~1.05 μm for CFRP and 0.47~0.62 μm for TC4. The results show that the hole wall surface roughness of both CFRP and TC4 using the adaptive machining method based on interface identification is better than that of the constant parameter machining method. The reason is that the adaptive machining method results in less wear and vibration of the cutting tool, which causes less scratching and damage to the machined hole wall.

Taking the last machined hole obtained from the experiment as an example, the entrance and exit qualities of the machined holes for each layer of material using the two machining experimental methods are listed in Table 5. Due to the more serious wear, edge disintegration, and chip adhesion of the cutting tool in the constant parameter machining method, which leads to a reduction in tool sharpness and cutting ability, the burr and delamination damage located at the entrance of the CFRP and the burr damage at the exit of the TC4 are more severe compared with the adaptive machining method. In addition, since the experimental parameters used in the constant parameter machining method are not suitable for TC4, which makes the cutting tool generate a large amount of heat during the machining of TC4, the excessively high temperature causes the softening and burning phenomenon of the resin at the exit of the CFRP. The entrance of the TC4 also appears to be blackened by burning, and the hole edge at the exit of the TC4 shows obvious burn marks.

### 4.4. Analysis of Cutting Tool Wear

The cutting tool wear is shown in Table 6. It is found that the constant parameter machining method has a wider wear area at the center and edge of the bottom edge of the cutting tool. Additionally, the microchipping, flaking, and chip adhesion phenomenon of the cutting edge are more serious and are accompanied by obvious bluish-black band burn traces. This is due to the fact that the machining parameters of this method are not applicable to the TC4 material in the bottom layer. When machining up to the TC4, the cutting force applied to the cutting tool increases, and the vibration is significantly intensified, which leads to the problems of increased wear and a higher local temperature of the cutting tool. The adaptive machining method based on interface identification ensures that each layer of material is machined at its respective suitable parameters, resulting in a significant improvement in the cutting tool wear.

## 5. Conclusions

In this paper, an adaptive machining method for the helical milling of CFRP/Ti stacks based on interface identification is proposed, a platform for helical milling is constructed, and experiments are carried out. Using a comparative analysis of the experimental results, the feasibility and accuracy of the proposed method are verified. The results show that the machining interface can be accurately identified using the proposed method, and the accuracy and quality of hole-making are significantly improved compared with the constant parameter machining method. The main conclusions of this paper can be summarized as follows:

(1) The machining mechanism of the helical milling method is investigated, the portable helical milling equipment is independently developed, and the spindle speed real-time feedback monitoring module is designed, so as to achieve the real-time reading of the spindle speed and the monitoring of the machining status of the equipment. Meanwhile, the interface identification method for the helical milling of CFRP/Ti stacks is proposed based on the characteristic that the spindle speed of the pneumatic spindle decreases with an increase in the torque applied to the spindle under the condition of constant input air pressure.
(2) A new adaptive machining method for the helical milling of CFRP/Ti stacks is further proposed based on the presented interface identification method. Using a machining experiment, theoretical calculation, and fitting analysis, the threshold values for the interface identification and adaptive adjustment of the machining parameters are determined, and the algorithmic logic and procedure of the method are established. The adaptive machining method for the helical milling of CFRP/Ti stacks based on interface identification takes full account of the complexity in the machining process of laminated components, without the need to clarify the specific thickness of each material layer. It is not sensitive to the thickness uniformity of the stacked layers and manufacturing errors, so the machining scope of application is much wider.
(3) Helical milling experiments of CFRP/Ti stacks are carried out, and analyzing the results, it is found that there is good agreement between the identified and monitored machined interface positions using the proposed method and the theoretical interface positions, which indicates that the proposed interface identification method has a high degree of accuracy. In addition, compared with the conventional constant parameter machining method, the proposed adaptive machining method for the helical milling of CFRP/Ti stacks based on interface identification results in higher hole-making accuracy and interlayer hole diameter consistency, as well as a significant improvement in the hole-making quality and wear of the cutting tool. This demonstrates the machining advancement and superiority of the proposed method.

## Figures and Tables

**Table 1:** Parameters of the model for each machining stage.

*y_a~g_*	Model Parameter *K_a~g_*	Model Parameter *N_a~g_*
*y_a_*	0	1585.68
*y_b_*	−137.65	1811.82
*y_c_*	0	1546.38
*y_d_*	−989.7	8824.45
*y_e_*	0	1364.15
*y_f_*	219.25	−1558.39
*y_g_*	0	1568.00

**Table 2:** Interface identification reference threshold values.

	CFRP Entry Interface (*n_Ce_*)	CFRP/Ti Transition Interface (*n*_*Te*_)	Ti Exit Interface (*n*_*To*_)
Threshold value	1575 r/min	1516 r/min	1402 r/min

**Table 3:** The experimental machining parameters.

	Constant Parameter Machining Method	Adaptive Machining Method Based on Interface Identification	
	Constant Parameters (*n_sc_*, *n_pc_*, *f_ac_*)	Parameters of CFRP layer (*n_sc_*, *n_pc_*, *f_ac_*)	Parameters of TC4 layer (*n_st_*, *n_pt_*, *f_at_*)
Spindle speed (r/min)	1600	1600	800
Revolution speed (r/min)	22	22	12
Axial feed speed (mm/min)	10	10	6

**Table 4:** Interface identification error results.

Hole-Making Number	CFRP Entry Interface Identification Errors (mm)	Transition Interface Identification Errors (mm)	TC4 exit Interface Identification Errors (mm)
1	0.05	0.06	−0.1
2	0.08	0.07	−0.14
3	0.09	0.05	−0.11
4	0.05	0.07	−0.12
5	0.07	0.06	−0.09
6	0.04	0.04	−0.16

**Table 5:** Entrance and exit quality of machined holes in each layer of material using two experimental methods.

	Entrance of CFRP	Exit of CFRP	Entrance of TC4	Exit of TC4
Constant parameter method	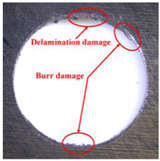	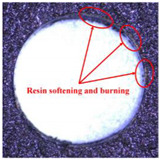	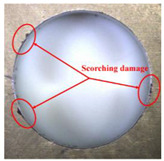	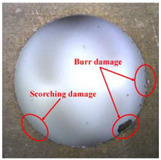
Adaptive machining method	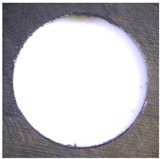	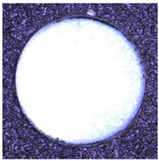	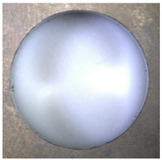	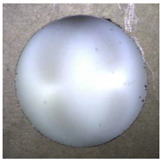

**Table 6:** The wear of the cutting tool.

	Cutting Tool	
Constant parameter method	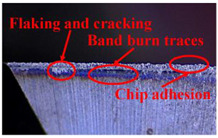	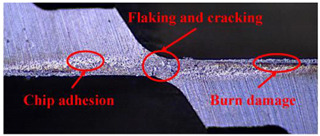
Adaptive machining method	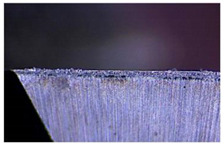	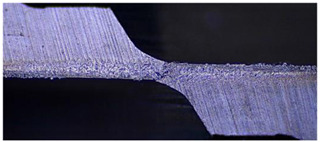

## Data Availability

Data are contained within the article.
